# The Book World of Medicine and Science

**Published:** 1898-05-14

**Authors:** 


### THE BOOK WORLD OF MEDICINE AND SCIENCE.

THE DIAGNOSIS OF DISEASE. By T. PORTER PARKINSON, M.D., M.R.C.P. Pp. 173. Price 4s. (London: Baillière, Tindall, and Cox. 1898.)

This little book is one of a type that is becoming regrettably familiar. It is intended "for the use of students and junior practitioners," and the author attempts the impossible task of suitably compressing the diagnosis of disease within 160 meagre pages. The result is inevitably a "cram book" pure and simple. Unfortunately, this book lacks the only merit a cram book can possess—accuracy, and we hope that in a future edition various errors which are apparently the result merely of looseness of expression will be rectified. In true Cheyne Stokes respiration there is no "interval of apnœa,' but a periodic variation in the amplitude of the respirations. The type of periodic breathing in which "intervals of apnœa" occur is another affair altogether, and was first described by Biot. Loss of movement and apical flattening (occurring as it does from loss of elasticity of lung tissue) is not only an advanced, but a very early sign of phthisis. There is no such thing known to physiologists as "low tension of blood"; "low arterial tension" or "low blood pressure" are correct phrases, but "low tension of blood" is meaningless. What "mitral convulsions" (p. 125) may be we cannot guess, and the table on the same page has nothing in the world to do, as the author states it has, with the seats of the lesions of hemiplegia. Lastly, every student who has read Dr. Buzzard's papers knows that disseminated sclerosis is not a disease chiefly of men, and that the knee-jerk is often absent. In books of this scope, above all things, accuracy and definitiveness of expression should be sought.

A HANDBOOK OF MIDWIFERY. By W. R. DAKIN, M.D., B.S., F.R.C.P., Obstetric Physician to and Lecturer on Midwifery and Diseases of Women at St. George's Hospital, Physician to the General Lying-in Hospital. (London: Longmans, Green, and Co., 1898. Price 18s.)

According to the author this is a work intended for students and junior practitioners, but we venture to think it will find a place in the libraries of, and be used by many senior members of the profession as a most useful work of reference on the matters with which it deals. The subject is arranged and treated in two divisions, the first having to do with the physiology and the second with the pathology of the various events connected with pregnancy, labour, and the puerperium. The writer has taken great pains to elucidate his meaning by the use of many excellent illustrations and diagrams. The greater number of these are stated to have been drawn at the time of writing especially to illustrate the text. They are thus, in most cases, diagrammatic rather than pictorial, being so drawn as to avoid the profusion of unnecessary detail so commonly found when plates are borrowed from other works, and they serve their purpose thoroughly well. The book is supplied with excellent indices both of the authors quoted and also of the subjects.

Appropriately enough the opening chapter is on embryology. This difficult subject is put in a readable form, while the details given are well up to date and, without entering deeply into the matter, contain the essentials necessary for any of the ordinary examinations in midwifery. The descriptions of the mechanism of labour and of the treatment of the various presentations, without containing much that is new, are clearly written and conveniently headed in a manner to impress the mind of the student. The chapter on puerperal fevers is good, the subject being more fully treated than in most text books.

The author regards these fevers from an ordinary common-sense surgical point of view, and entirely discards the notion that they require for their explanation any special vulnerability in the lying-in woman depending on the blood condition. "The affections of the puerperal state may, in fact, be considered as ordinary affections attacking a patient in a traumatic condition." "The sooner we lose the idea that the puerperal woman differs in any way which suggests the assumption of a temporary diathesis from a non-puerperal woman who has similar breaches of surface the better we shall understand the true source and the extrinsic nature of all her diseases." This puts midwifery in proper line with surgery. Under the head of "Pelvic Measurements" the author gives the conjugata vera as four and a quarter inches, but we are surprised to find lower down that he says "it will be found convenient for the sake of getting a whole number to deal with to call it four inches"; but surely if the student is to remember the number at all, it is much better for him to recollect the correct one. On page 185 a list is given of articles to be obtained by the mother or nurse, and kept in readiness for the doctor; amongst other things mentioned we find four ounces of chloroform, but we doubt if it is usual, and we are sure it is not desirable, for the expectant mother to provide and keep a dangerous drug of this description. These, however, are trivial blemishes. With few exceptions the book is decidedly good, its teaching is sound, and we can confidently recommend it to the student.

